# Local Wnt3a treatment restores bone regeneration in large osseous defects after surgical debridement of osteomyelitis

**DOI:** 10.1007/s00109-020-01924-9

**Published:** 2020-05-18

**Authors:** Johannes Maximilian Wagner, Felix Reinkemeier, Mehran Dadras, Christoph Wallner, Julika Huber, Alexander Sogorski, Maxi Sacher, Sonja Schmidt, Marius Drysch, Stephanie Dittfeld, Mustafa Becerikli, Kathrin Becker, Nicole Rauch, Marcus Lehnhardt, Björn Behr

**Affiliations:** 1 University Hospital BG Bergmannsheil Bochum, Bürkle-de-la-Camp Platz 1, 44789 Bochum, Germany; 2 Poliklinik für Kieferorthopädie, University Hospital Düsseldorf, Düsseldorf, Germany

**Keywords:** Bone, Osteomyelitis, Wnt3a, Canonical Wnt-pathway, Bone regeneration

## Abstract

**Abstract:**

Impaired bone homeostasis caused by osteomyelitis provokes serious variations in the bone remodeling process, thereby involving multiple inflammatory cytokines to activate bone healing. We have previously established a mouse model for post-traumatic osteomyelitis and studied bone regeneration after sufficient debridement. Moreover, we could further characterize the postinfectious inflammatory state of bony defects after debridement with elevated osteoclasts and decreased bone formation despite the absence of bacteria. In this study, we investigated the positive effects of Wnt-pathway modulation on bone regeneration in our previous established mouse model. This was achieved by local application of Wnt3a, a recombinant activator of the canonical Wnt-pathway. Application of Wnt3a could enhance new bone formation, which was verified by histological and μ-CT analysis. Moreover, histology and western blots revealed enhanced osteoblastogenesis and downregulated osteoclasts in a RANKL-dependent manner. Further analysis of Wnt-pathway showed downregulation after bone infections were reconstituted by application of Wnt3a. Interestingly, Wnt-inhibitory proteins Dickkopf 1 (DKK1), sclerostin, and secreted frizzled protein 1 (sFRP1) were upregulated simultaneously to Wnt-pathway activation, indicating a negative feedback for active form of Beta-catenin. In this study, we could demonstrate enhanced bone formation in defects caused by post-traumatic osteomyelitis after Wnt3a application.

**Key messages:**

- Osteomyelitis decreases bone regeneration
- Wnt3a restores bone healing after infection
- Canonical Wnt-pathway activation with negative feedback

**Electronic supplementary material:**

The online version of this article (10.1007/s00109-020-01924-9) contains supplementary material, which is available to authorized users.

## Background

One of the main evolutionary driving forces in humans is the capability to combat infections. In this context, bone infections are of particular interest and need special attention as they pose one of the biggest problems in orthopedic surgery especially after open fractures of long bones [1]. The incidence of osteomyelitis (OM) in elective bone surgery is considered to be 1–5%. However, OM still occurs in 5–30% of open fractures, depending mainly on the severity of the fracture [1].

In the presence of necrotic bone material, the effectiveness of antibiotic therapy can be dramatically reduced due to biofilm formation [2]. The mainstay of treatment is therefore adequate surgical debridement, thus removing all purulent tissue.

In previous findings, we could demonstrate the essential status of surgical debridement versus antibiotic treatment alone in a murine tibia defect model [3], as eradication of bacteria could only be achieved by surgical intervention. Depending on the extent of infection, large bony defects can remain. Moreover, the bone in the vicinity still shows a dramatically reduced regenerative capacity despite complete absence of bacteria [3] after sufficient debridement. Accordingly, a further own study could identify an ongoing inflammatory reaction which leads to an upregulation of cytokines and activation of the innate immune system verified by elevated B cells [4].

In a curative approach, the bone needs to be reconstructed or amputated. Immobility and mortality increases proportional to the level of amputation, given the higher energy and oxygen demand post-amputation [5]. For this reason, every effort should be attempted to limit the amount of bone loss by means of debridement and achieve bony reconstruction of the defects.

In bone biology, bone formation and resorption are highly complex regulated processes, which need to be balanced during physiological bone regeneration. Elevated bone resorption is a distinct feature of osteomyelitis, which leads to bone erosion quite similar to processes in rheumatoid arthritis [6]. In this context, plenty of mechanisms in bone inflammation have already been postulated, many involving the activation of the receptor activator nuclear factor-κB ligand (RANKL) which induces nuclear factor of activated T cells, cytoplasmic 1 (NFATc1), resulting in osteoclastogenesis [7]. Our recent work indicated elevated bone resorption after successful debridement of bone infections in mice [4]. This was mainly caused by an elevated osteoclastogenesis occurring together with an upregulation of RANKL activity. Interestingly, TNF-α, which is known to be a main activator of RANKL pathway [8], was not involved in this inflammatory process. Regarding increased bone resorption, distal-less homebox protein 5 (DLX-5) is known to be crucial for osteoblast-osteoclast interaction and osteoclast regulation [9] and decreased levels of DLX-5 are associated to an increased bone resorption.

Wnt-pathway is an important pathway for plenty of different regenerative processes.

In canonical Wnt-pathway, Beta-catenin is formed continuously. While the Wnt-pathway is inactivated, a complex consisting of axis inhibition protein (Axin), adenomatous polyposis coli (APC), glycogen synthase kinase 3 beta (GSK-3β), and casein kinase 1 alpha (CK1α) phosphorylates and thereby inactivates Beta-catenin [10, 11]. Pathway activation leads to an increased recruitment of Disheveled (Dvl), which in turn inhibits the inactivation complex [12]. Activated Beta-catenin translocates into nucleus and acts as a co-activator of transcription factor (T Cell Specific, HMG-Box) (TCF)/lymphoid enhancer-binding factor (LEF) [13].

It has already been shown that Wnt3a is a strong promoter of bone formation in differentiated cells [14–20]. Moreover, Wnt-pathway is known to increase angiogenesis in various tissues and organs, mainly via increased expression of vascular endothelial growth factor (VEGF) [21]. However, studies investigating Wnt-pathway in the setting of inflammatory diseases are sparse.

In the light of altered bone regeneration after post-traumatic osteomyelitis, we sought to improve bone healing through direct activation of canonical Wnt-pathway.

Thus, we applied Wnt3a locally after debridement in our established murine bone infection model [3]. Decreased bone regeneration after infection could be restored via increased osteogenesis and inhibition of osteoclast activity.

## Methods

### Mouse osteomyelitis model

Animals were housed and caged individually with free access to water and food under specific pathogen-free conditions. C57BL/6 J male and female mice, 12 weeks old with an average weight of 25 g, were used for this project. Surgical steps were performed, as previously described [3] in our previously established animal model. Briefly, a skin incision was placed over the proximal medial tibia, and a hole (1 mm in diameter) was drilled into the proximal medial tibia after retraction of the tibialis anterior muscle. Thereafter, *S. aureus* in a concentration of 1000 units per μl was injected into the medullary cavity of the tibia. Infection of the tibia was performed in all experimental animals in this study except for animals in a group of uninfected control. Thereafter, the muscle was reapproximated and the wound closed.

Two weeks after *Staph. aureus* inoculation, a second surgery for sufficient debridement was performed. The skin incision was placed once again over the proximal medial tibia exposing the bone defect. Then, infected lytic bone tissue was debrided and completely removed, followed by rinsing of debrided defects with isotone sodium chloride solution.

Thereafter, 1-μg Wnt3a (recombinant mouse Wnt3a, Peprotech, Rocky Hill, USA) in 1 ml was placed into the bony defect on a collagen sponge. As a control, 1 ml of PBS was administered to the bone defect on a collagen sponge as well. Moreover, infected animals (OM) and uninfected control (control) received no treatment.

Mice were sacrificed 3 and 7 days after debridement which represents an early and late time point during bone regeneration, according to the experiences of Colnot et al. [22].

### Protein isolation

For protein isolation, only the bony defect of 9 animals per group was used, removing all of the remaining parts of the bone except for the region of interest.

Thereafter, the bony defect was snap frozen after harvest and stored at − 80 °C. For further processing of the specimens, bony defects were frozen in liquid nitrogen and pestled into smaller bone fragments. Then, bone fragments were collected and homogenized in lysis buffer, containing protease inhibitors (aprotinin, leupeptin, pepstatin A, and phosphatase inhibitor), until lysis was completed. Then, cellular debris was removed by centrifugation, and isolated protein was stored at − 80 °C for further experiments.

### Western blot

Isolated protein was combined and mixed with Laemmli sample buffer. After denaturation at 95 °C, samples were directly loaded onto the SDS PAGE. Fifteen percent polyacrylamide gels were used for electrophoresis of 30-μg total protein per lane. Protein was transferred to a nitrocellulose membrane using wet transfer method before membranes were blocked with 3% bovine serum albumin to prevent unspecific binding. After blocking, membranes were washed again in TBS-T to remove remaining blocking solution. Hereafter, membranes were incubated with primary antibodies against Beta-catenin, GSK-3β (cat. nr. 19807S, 5558S) (cell signaling technology, Danvers, USA), RANKL, Runx2, DKK1, sclerostin, sFRP1, and osteoprotegerin (cat.nr. ab45039, ab23981, ab61275, ab63097, ab4193, ab183910) (Abcam, Cambridge, UK) overnight at 4 °C, followed by washing and incubation with HRP-conjugated secondary antibody (Thermo Fisher Scientific, Waltham, USA; Santa Cruz Biotechnologies, Dallas, USA). Both antibodies were diluted in BSA. Proteins were detected for 30–60 s by enhanced chemoluminescence using Kodak Image Station 4000 mm (Kodak, Rochester, USA) according to standard protocol [23].

### Micro-computed tomographic analysis

Bone specimens of 3 animals per group were scanned with a μCT device (Viva CT 80; Scanco Medical AG, Brüttisellen, Switzerland) operated at 70 kVp, 114 μA, 8 W, 31.9-mm FOV, an integration time of 1167 ms, and 2x frame averaging. The data sets were reconstructed into 3D volumes with an isotropic nominal resolution of 15.6-μm voxel size.

### Image processing

Further processing of the scanned images was performed using μ-CT Evaluation Software Program V6.5 (Scanco Medical AG, Brüttisellen, Switzerland). A standardized cylindrical volume of interest with 16.84 mm in diameter was placed within the defect site, defining the region of interest. Thereafter bone volume to total volume (BV/TV) was assessed according to the guidelines for assessment of bone microstructures using μCT (Bouxsein, Guidelines for assessment of bone microstructure in rodents using micro-computed tomography 2010).

### Histology, immunohistochemistry, and immunofluorescence

For all histological procedures, tibiae of 6 mice per group were taken and fixed in 4% paraformaldehyde solution overnight, decalcified in 19% EDTA solution, and finally paraffin embedded. Thereafter, tibiae were longitudinally sectioned at 9 μm. Following, aniline blue staining was performed as previously described [24]. Images were taken with Zeiss Axivert 100 (Zeiss, Oberkochen, Germany). Quantification of aniline blue positive pixels was performed in Adobe Photoshop placing a 2000 × 2000 pixel dimension selection box over the entire defect area. The magic wand tool (tolerance 60%; non-contiguous) was used to mark aniline blue stained osteoid formation semi-automatically. Thereafter, cortical bone was deselected manually. The highlighted pixels reliably corresponded to the new bone formation area.

Furthermore, TRAP-staining was performed with TRAP Kit (Sigma-Aldrich, St. Louis, USA) according to the manufacturers’ instruction.

Additionally, immunohistochemical staining were performed using Vectastain ABC Kit (Vector Laboratories, Burlingame, USA) with primary antibodies against osteocalcin, PCNA (cat. nr. sc-30,045, sc-7907) (Santa Cruz Biotechnologies, Dallas, USA) and CD31 (cat. nr. 553,700) (BD Biosciences, Franklin Lakes, USA). After deparaffinization and rehydration of bone sections after standard protocols, antigen demasking was performed using 1% proteinase K solution. Thereafter, specimens were incubated with 3% hydrogen peroxide solution to quench endogenous peroxidase activity. Thereafter, specimens were blocked with normal blocking serum to prevent unspecific binding of primary antibody that was subsequently applicated and incubated overnight at 4 °C. Following, secondary antibody conjugated to horseradish peroxidase (HRP) was used, and staining reaction was performed by the use of NovaRED (HRP) Peroxidase Substrate Kit (Vector Laboratories, Burlingame, USA).

For immunofluorescent staining, primary antibodies against Runx2 (cat. nr. sc-10,758) (Santa Cruz Biotechnologies, Dallas, USA), Beta-catenin, GSK-3β (cat. nr. 19807S, 5558S) (cell signaling technology, Danvers, USA) were used. Initial steps were carried out similar to immunohistochemical staining until application of primary antibody. Thereafter, samples were incubated with secondary antibody conjugated to Alexa Fluor594 (Thermo Fisher Scientific, Waltham, USA). Images were taken with Zeiss Axioplan microscope. Quantification of immunohistochemistry was performed selecting a region of interest of 2000 × 2000 Px and automatic selection via magic wand tool (tolerance 60%; noncontiguous). Quantification of TRAP stain pixels was carried out in relation to pixels of bone surface according to standard protocol [25].

### Statistics

Results are presented as mean ± standard error of the mean (SEM) of at least three independent experiments. Shapiro-Wilk test was used to check for normal distribution. Additionally, f-test was used to examine variances of both groups.

*p* values were calculated by student’s *t* test comparing two groups. For post hoc comparisons, Tukey’s test was used. Statistical significances were set at a *p* value < 0.05.

## Results

### Canonical Wnt-pathway is downregulated in post-infectious inflammatory state

**Fig. 1:**
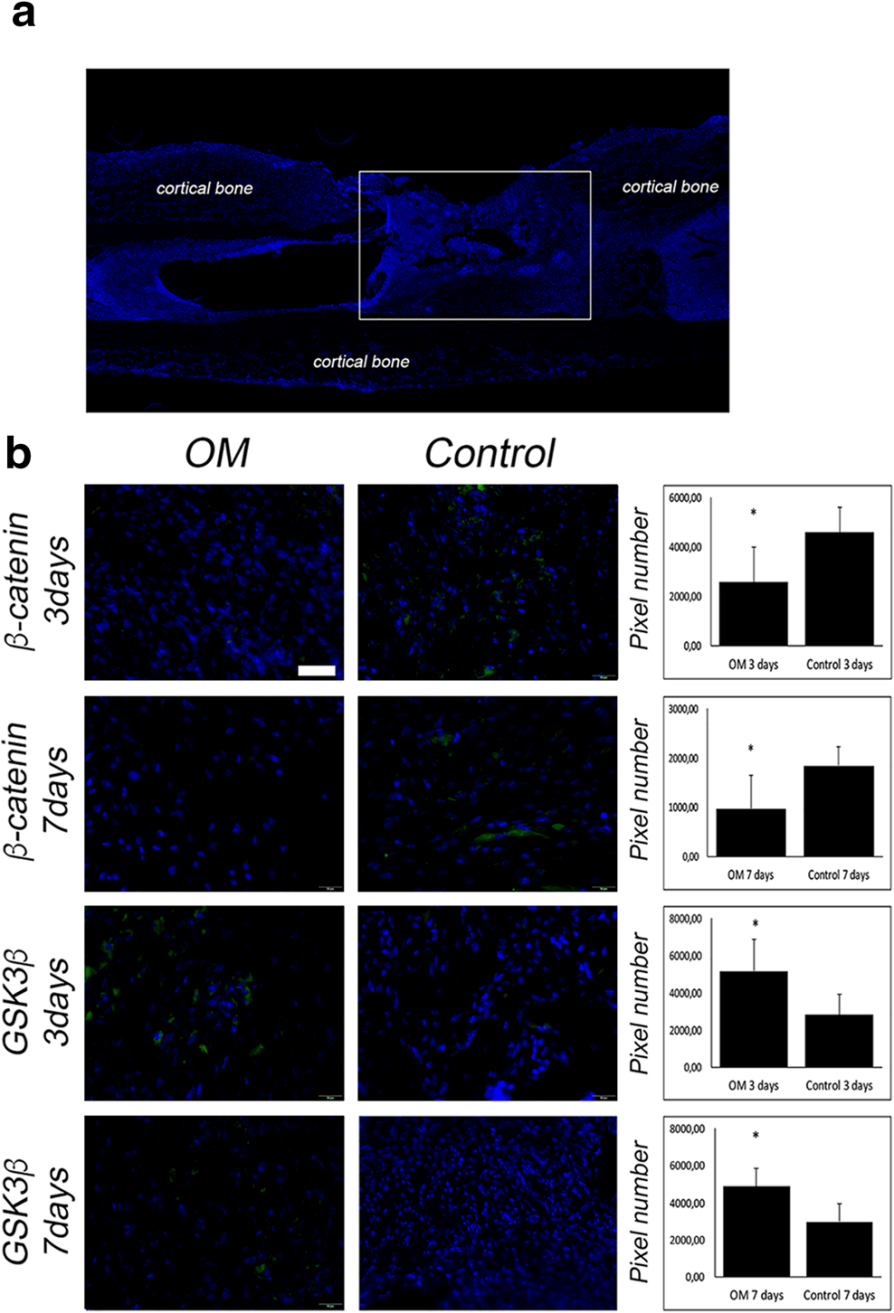
Postinfectious inflammatory state of osteomyelitis impairs canonical Wnt-pathway. (**A**) Overview of immunofluorescent images of the tibial bone, DAPI-stain (blue). The white square marks the region of interest of all immunoflourescent and immunohistochemical staining. (**B**) Immunoflourescent staining against β-catenin (green), GSK-3β (green) and DAPI (blue) of infected and debrided animals (OM) and uninfected control animals (Control). Y-axis stands for pixel number of stained pixel quantification. Scale bar represents 20 μm; *p* value, * < 0.05

In order to check canonical Wnt-pathway activity, immunoflourescent staining against glycogen synthase kinase (GSK-3β) and Beta-catenin were performed. Infected animals, which underwent no further treatment showed enhanced signaling of GSK-3β and decreased Beta-catenin in comparison with the uninfected control, indicating decreased activity of the canonical Wnt-pathway due to infection (Fig. 1B).

### Wnt3a application enhances osteogenesis and angiogenesis after osteomyelitis

**Fig. 2:**
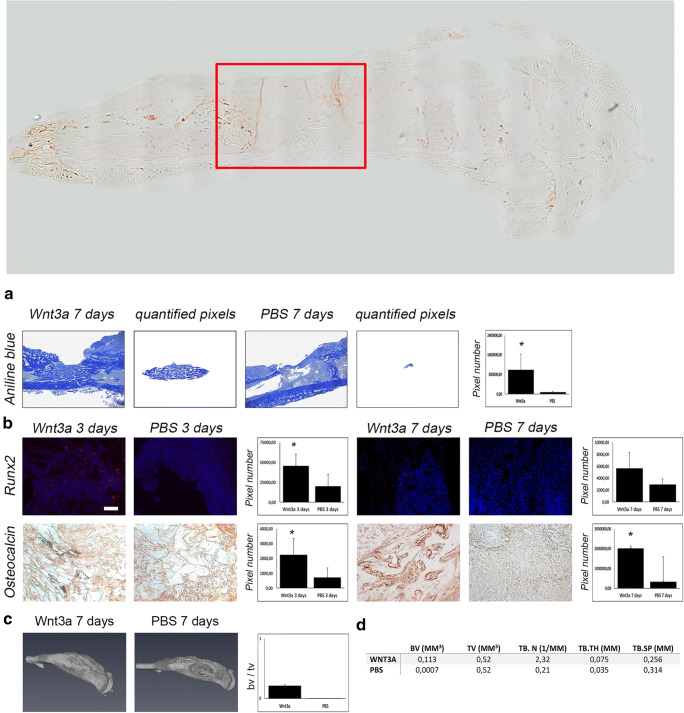
Enhanced osteogenesis and osteoblastogenesis due to Wnt3a treatment. Overview of tibial defect and region of interest (red square) of immunofluorescent and immunohistochemical staining (above). (**A**) Aniline blue staining and (**B**) immunoflourescent staining against Runx2 (red) and DAPI (blue) and immunohistochemical staining against osteocalcin (red) of infected animals treated by Wnt3a or PBS. Y-axis stands for pixel number of stained pixel quantification. Scale bar aniline blue represents 400 μm; Scale bar Runx2 and osteocalcin represents 100 μm; *p* value, * < 0.05. (**C**) μCT scans of Wnt3a and PBS group tibia. Y-axis stands for bone volume to total volume (bv/tv) measured within defect site. (**D**) bone volume (BV) in mm^3^, total volume (TV) in mm^3^, trabecular number (TB.N) in 1/mm, trabecular thickness (TB.TH) in mm, and trabecular separation (TB.SP) in mm of region of interest of μCT scans of WNT3a and PBS group

**Fig. 3:**
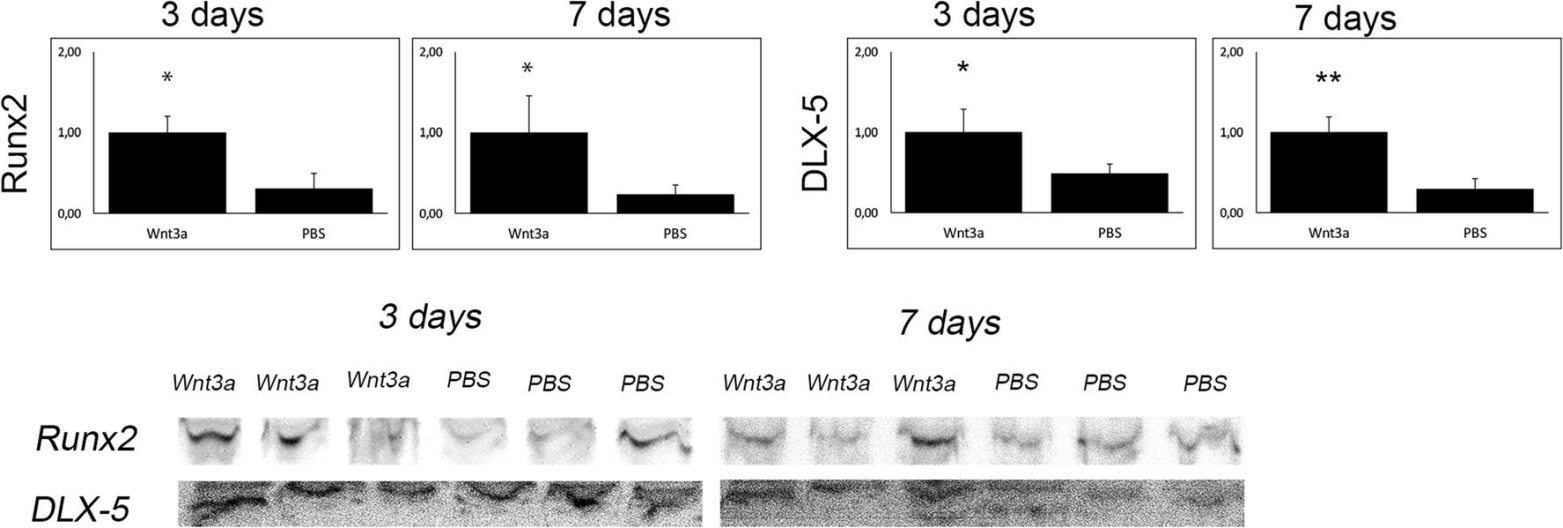
Western blots verified upregulated osteoblastogenesis due to Wnt3a activation. Data obtained from western blots of WNT3a and PBS group after 3 and 7 days and cropped images of western blots against markers for osteoblastogenesis (Runx2, DLX5). *p* value, * < 0.05, ** < 0.01

**Fig. 4:**
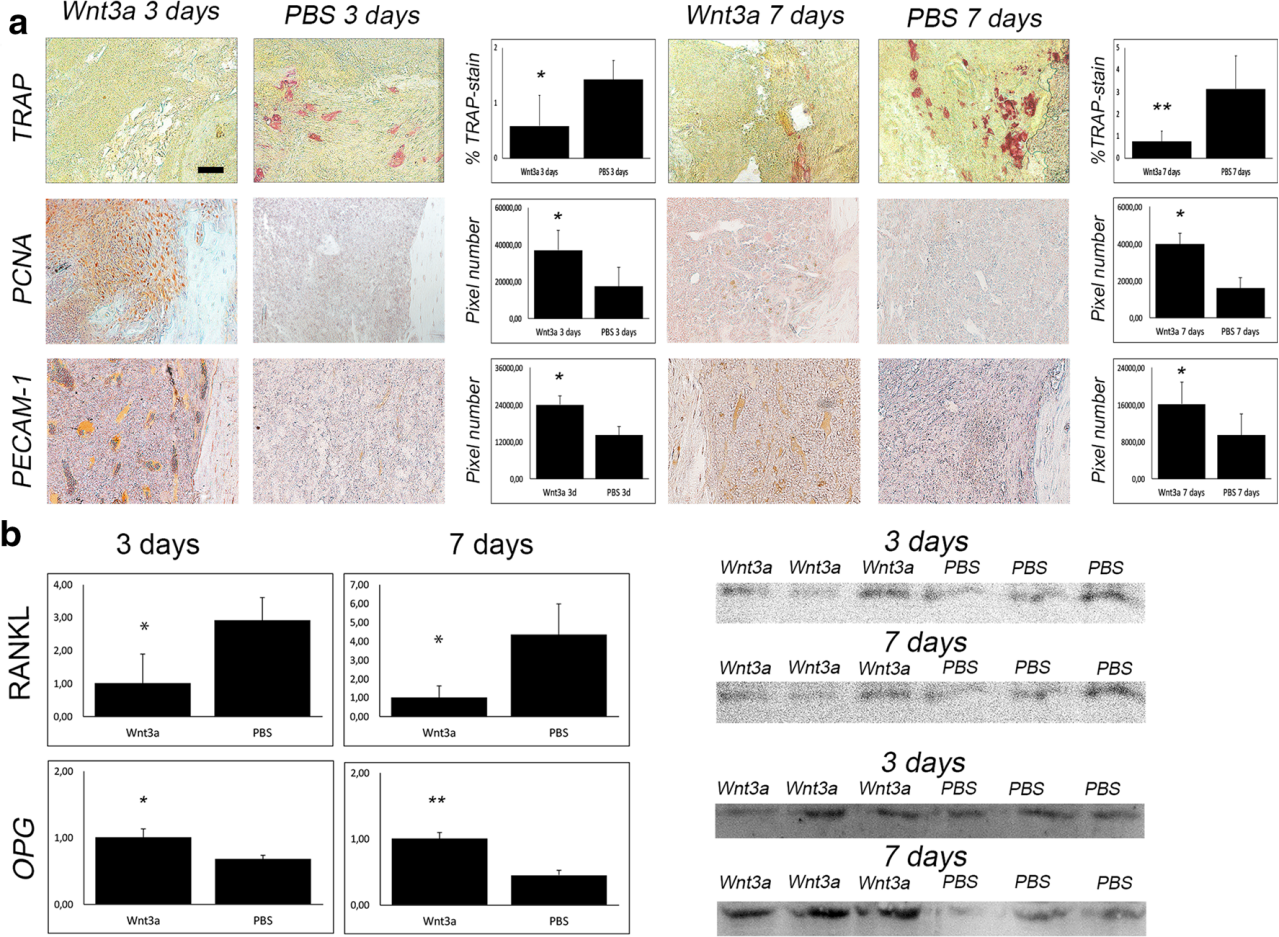
Wnt-pathway activation leads to diminished osteoclasts and increased angiogenesis and proliferation. (**A**) TRAP staining and immunohistochemical staining against PCNA (red) and PECAM-1 (red) of infected Wnt3a and PBS treated animals 3 and 7 days after treatment. Y-Axis stands for pixel number of stained pixel quantification. Scale bar represents 100 μm; *p* value, * < 0.05. (**B**) Western blot data and cropped images of Wnt3a and PBS group after 3 and 7 days of markers concerning osteoclastogenesis (RANKL, OPG). *p* value, * < 0.05, ** < 0.01

After application of Wnt3a, our first interest was to evaluate new bone formation upon treatment. Aniline blue staining (Fig. 2A) and μ-CT analysis (Fig. 2C and D) revealed significantly increased osteogenesis 7 days after Wnt3a application. Accordingly, elevated osteoblastogenesis and mature osteoblasts could be observed in runt-related transcription factor 2 (Runx2) and osteocalcin staining (Fig. 2B), as well as western blots (Fig. 3). Moreover, distal-less homebox protein 5 (DLX-5), which is known to be a potent modulator of Runx2 and thereby osteoblast differentiation, was also upregulated by Wnt3a application (Fig. 3). Furthermore, staining detecting Proliferating-Cell-Nuclear-Antigen (PCNA) revealed enhanced proliferation after Wnt3a treatment (Fig. 4A). Interestingly, staining against PECAM-1 showed increased angiogenesis after activation of canonical Wnt-pathway (Fig. 4A).

### Osteoclast activity can be decreased by canonical Wnt-pathway activation

Elevated osteoclast activity was one of the main reasons for altered bone regeneration during inflammatory state after post-traumatic osteomyelitis. As Wnt-pathway activation is known to diminish osteoclast activity^20^, we were interested to investigate osteoclasts after activation of canonical Wnt-pathway.

Subsequently, after Wnt3a application, animals showed less osteoclast activity in TRAP-staining (Fig. 4A) accompanied by decreased levels of receptor activator of NF-κB Ligand (RANKL) and elevated levels of osteoprotegerin in western blot (Fig. 4B).

### Wnt-pathway inhibitors are upregulated upon Wnt3a application

**Fig. 5:**
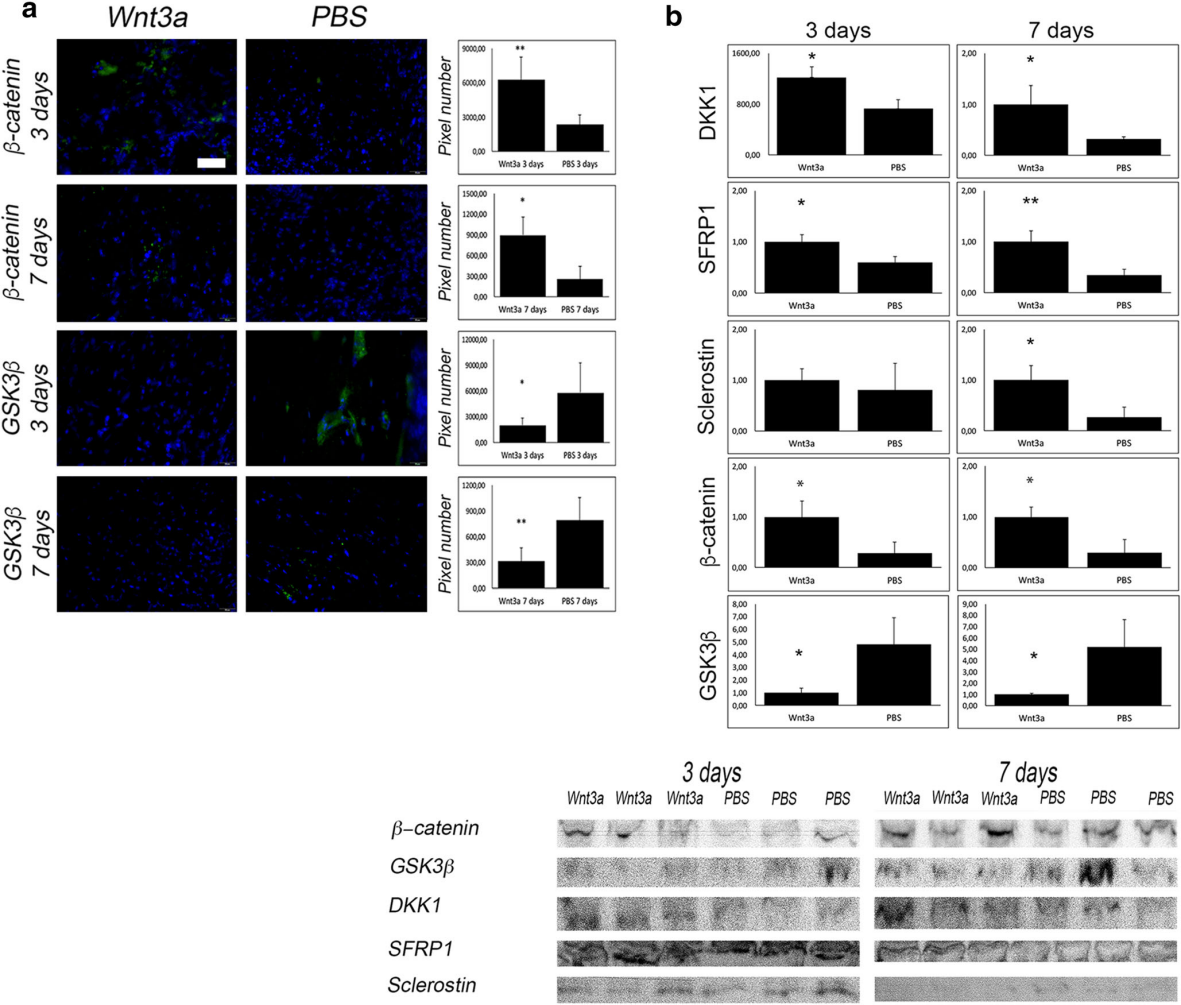
Wnt3a application activates Wnt-pathway. (**A**) Immunoflourescent staining against Beta-catenin (green) and GSK-3β (green) and DAPI (blue) of infected and Wnt3a and PBS treated animals. Y-axis stands for pixel number of stained pixel quantification. Scale bar represents 20 μm; *p* value, * < 0.05, ** < 0.01. (**B**) Western blot data and cropped images of Wnt3a and PBS group after 3 and 7 days of Wnt-pathway activity markers (Beta-catenin, GSK-3β, DKK1, SFRP1, sclerostin). *p* value, * < 0.05, ** < 0.01

Addition of Wnt3a subsequently restored canonical Wnt-pathway activity, whereas decreased Wnt-activity could be seen in PBS-treated control animals, similar to infected animals which received no further treatment (Fig. 5A).

In accordance, western blot showed similar findings of GSK-3β and Beta-catenin levels (Fig. 5B).

Besides Wnt-pathway activity, our interest was to elucidate the orchestration of activation and inhibition of the Wnt-pathway. Interestingly, inhibitory proteins, Dickkopf-related protein (DKK1), secreted frizzled-related protein 1 (sFRP1), and sclerostin all seemed to be upregulated in the Wnt3a group (Fig. 5B). Except sclerostin, which was only regulated on day 7, all of these factors were elevated on both day 3 and 7.

## Discussion

It is well accepted that inflammatory processes diminish regenerative healing capacity. Especially in bone infections, this is a common problem of orthopedic surgery [26]. Based on previous studies [3, 4], this work aimed to improve bone healing via activation of canonical Wnt-pathway and reveal Wnt-dependent mechanisms of bone formation and resorption related to bone infection.

Our focus was to evaluate the regenerative capacity of Wnt3a-treated mice in a murine post-traumatic osteomyelitis model. Histology and western blots revealed elevated bone formation, osteoblastogenesis, and angiogenesis. Interestingly, these differences could be seen throughout the observation period, during early and late bone regeneration, despite a single Wnt3a treatment at the beginning.

Osteogenic markers Runx2 and DLX-5 were significantly upregulated in Wnt3a group, suggesting a regulation via Wnt-pathway. In this context, direct activation of Runx2 by Beta-catenin has already been demonstrated in various settings [27–29] and proved to be an effective treatment strategy to enhance bone regeneration in long bone fractures [27].

DLX-5 is known to be crucial for endochondral ossification and moreover regulates osteogenic factors like Runx2 and Osterix [30–32]. Typically activated by the bone morphogenetic protein (BMP) pathway [30, 33], only little is known about Wnt pathway interaction. Elevated BMP-2 and thereby DLX-5 levels have been described after canonical Wnt-pathway activation [34].

Similarly to our observations, activation of canonical Wnt-pathway is known to be a strong promoter of angiogenesis during physiological and pathological conditions, regulating VEGF and other factors crucial for angiogenesis [21].

Concerning bone resorption, we could observe a markedly diminished osteoclasts activity presumably regulated by the RANKL/OPG axis via activation of canonical Wnt-pathway. Especially patients with inflammatory joint diseases like rheumatoid arthritis suffer from excessive RANKL expression and enhanced bone resorption [35], making it therefore a therapeutical target in these patients [36]. Moreover, a deregulation of the RANKL/OPG axis is consistent to our previous work, characterizing bone healing after osteomyelitis, revealing a post-infectious inflammatory state with elevated osteoclast activity and diminished osteoblastogenesis [4].

It is well accepted that Wnt3a and other activators of canonical Wnt-pathway are strong promoters of bone regeneration [14–20], enhancing osteoblastogenesis and thereby bone formation. Besides that, bone resorption is known to be affected by canonical Wnt-pathway, decreasing osteoclast activity via modulation of osteoclast progenitor cell differentiation and RANKL/OPG axis [37–39].

To verify canonical Wnt-pathway activation, the active form of Beta-catenin and GSK-3β was investigated. In Wnt3a group, Wnt-pathway activation was markedly increased throughout the observation period. However, only little is known about Wnt-pathway activation in the setting of inflammatory response after bone infection.

It has been shown that osteoblasts in the vicinity of inflamed joints in rheumatoid arthritis (RA) showed less osteogenic markers while upregulation of Wnt inhibitors DKK1 and Frizzled-related proteins [40].

Moreover, blocking antibodies of DKK-1 promotes bone formation and prevents bone erosion [41]. Interestingly, activated canonical Wnt-signaling might also have immunomodulatory roles as activated Beta-catenin can reduce bacteria-induced inflammation [42]. In a *Salmonella* infection model, constitutive activation of Beta-catenin (and thereby canonical Wnt-signaling) showed inhibition of NF-κB-activity [43].

Wnt-pathway is a highly complex-regulated network involving different agonists and inhibitory factors. Some of the most important inhibitory factors, namely DKK1, sFRP1, and sclerostin, were all elevated after Wnt3a treatment.

DKK1 is a direct inhibitor of canonical Wnt-pathway. Interestingly, a negative feedback loop, increasing the expression of DKK1 initiated via Beta-catenin is well known [44–46]. Moreover, activation of Wnt/Beta-catenin pathway by neutralizing antibodies against sclerostin was accompanied by increased levels of sclerostin and DKK1 [47, 48] This leads to the assumption of a negative-feedback between canonical Wnt-pathway and sclerostin as well and thus limiting Wnt-driven bone formation. Consistently, a dual inhibition of sclerostin and DKK1 leads to increased bone formation in comparison with inhibition of each one of them separately [48]. For example, in patients with type 2 diabetic osteoporosis, a close link of bone loss to the canonical Wnt pathway with increased expression of DKK1 and sclerostin became evident in a recently published study [49]. Within this context, upregulated levels of sFRP1 are also associated with different metabolic bone disorders including osteoporosis, mainly because of Wnt/Beta-catenin inhibition [50]. Originally, being identified as a Wnt inhibitor, more recent studies revealed a more differentiated role of sFRP1 in Wnt signaling. Depending on the cellular context, concentration and expression patterns of Fzd receptors sFRP1 could either promote or inhibit Wnt/Beta-catenin pathway. Interestingly, a direct link between Hedgehog signaling pathway and Beta-catenin was described. Downregulation of the Hedgehog signaling via glioma-associated oncogene homolog 3 (GLI3) leads in turn to a downregulation of sFRP1 [51]. Accordingly, the upregulation of sFRP1 after Wnt3a application in this work seems striking. However, we propose another regulatory mechanism leading to upregulation of sFRP1 in this case, as the regulation of sFRPs in Wnt pathway is not fully understood and needs to be further investigated. In fact, sFRP1 has protective regulatory Wnt-related effects against carcinomas in various cell types, making this assumption more likely [50].

The anabolic functions of Wnt/Beta-catenin pathway could restore the bone healing deficiencies after post-traumatic osteomyelitis in long bone. Local Wnt3a application led to increased bone formation, affecting both osteoblasts via Runx2 and DLX-5 upregulation and osteoclasts shifting RANKL/OPG-axis. Moreover, the osteogenic capacity of canonical Wnt-pathway activation was regulated by elevated Wnt inhibitors DKK1 and sclerostin.

Activating Wnt-pathway could promote bone regeneration of patients with large bony defects after radical debridement of osteomyelitis. To date, commercially available antibodies against sclerostin exist, being used to increase bone mass in postmenopausal osteopenia [52].

## Conclusions

The findings of this study could show the beneficial effects of Wnt3a application on bone regeneration after post-traumatic osteomyelitis. Subsequently, activation of canonical Wnt-pathway leads to increased bone formation and osteoblastogenesis, whereas osteoclast activity could be decreased. Interestingly, activation of Wnt/Beta-catenin pathway elevated pathway inhibitors DKK1, sclerostin, and sFRP1. Thus, a negative feedback of active Beta-catenin could be assumed.

## Electronic supplementary material

Figure S1 (PNG 2896 kb)

High resolution image (TIF 3484 kb)

## Abbreviations

OM: osteomyelitis
RANKL: receptor activator nuclear factor-κB ligand
OPG: osteoprotegerin
NFATc1: nuclear factor of activated T cells, cytoplasmic 1
TNF-α: tumor necrosis factor alpha
Axin: axis inhibition protein
APC: adenomatous polyposis coli
GSK-3β: glycogen synthase kinase 3 beta
CK1α: casein kinase 1 alpha
Dvl: Disheveled
Runx 2: runt-related transcription factor 2
DKK1: Dickkopf-related protein 1
PCNA: Proliferating-Cell-Nuclear-Antigen
sFRP1: secreted frizzled-related protein 1
DLX-5: distal-less homebox protein 5
PECAM-1: platelet endothelial cell adhesion molecule 1
LRP: low-density lipoprotein receptor related protein
