# Correction to: Local Wnt3a treatment restores bone regeneration in large osseous defects after surgical debridement of osteomyelitis

**DOI:** 10.1007/s00109-021-02144-5

**Published:** 2021-10-19

**Authors:** Johannes Maximilian Wagner, Felix Reinkemeier, Mehran Dadras, Christoph Wallner, Julika Huber, Alexander Sogorski, Maxi Sacher, Sonja Schmidt, Marius Drysch, Stephanie Dittfeld, Mustafa Becerikli, Kathrin Becker, Nicole Rauch, Marcus Lehnhardt, Björn Behr

**Affiliations:** 1 University Hospital BG Bergmannsheil Bochum, Bürkle-de-la-Camp Platz 1, 44789 Bochum, Germany; 2 Poliklinik Für Kieferorthopädie, University Hospital Düsseldorf, Düsseldorf, Germany

## Correction to: Journal of Molecular Medicine (2020) 98:897–906 https://doi.org/10.1007/s00109-020–01,924-9

The article Local Wnt3a treatment restores bone regeneration in large osseous defects after surgical debridement of osteomyelitis by Johannes Maximilian Wagner, Felix Reinkemeier, Mehran Dadras, Christoph Wallner, Julika Huber, Alexander Sogorski, Maxi Sacher, Sonja Schmidt, Marius Drysch, Stephanie Dittfeld, Mustafa Becerikli, Kathrin Becker, Nicole Rauch, Marcus Lehnhardt and Björn Behr, was originally published online on 18 May 2020 without open access. With the author(s)’ decision to opt for Open Choice the copyright of the article changed on 21 September 2021 to © The Author(s) 2021 and the article is forthwith distributed under a Creative Commons Attribution 4.0 International License, which permits use, sharing, adaptation, distribution and reproduction in any medium or format, as long as you give appropriate credit to the original author(s) and the source, provide a link to the Creative Commons licence, and indicate if changes were made. The images or other third party material in this article are included in the article’s Creative Commons licence, unless indicated otherwise in a credit line to the material. If material is not included in the article’s Creative Commons licence and your intended use is not permitted by statutory regulation or exceeds the permitted use, you will need to obtain permission directly from the copyright holder. To view a copy of this licence, visit http://creativecommons.org/licenses/by/4.0.

The Original article has been corrected.

