# Supplementary figures and images for: Local Wnt3a treatment restores bone regeneration in large osseous defects after surgical debridement of osteomyelitis
Source: J Mol Med (Berl). 2020 May 18;98(6):897–906. doi: 10.1007/s00109-020-01924-9 (PMC8526481; doi:10.1007/s00109-020-01924-9)

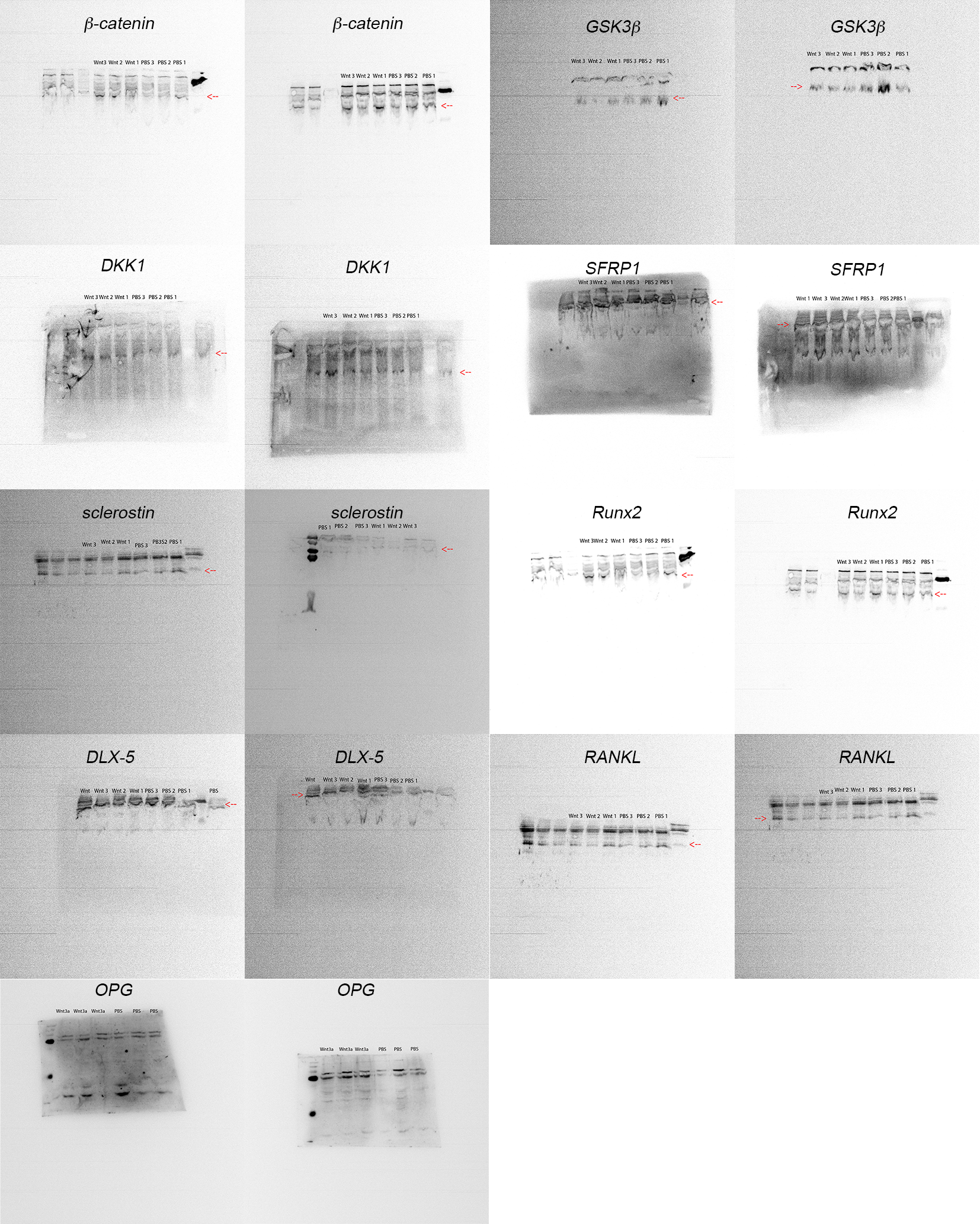

Supplement: Supplementary file 1 — (PNG 2896 kb) [file 109_2020_1924_Fig6_ESM.png]

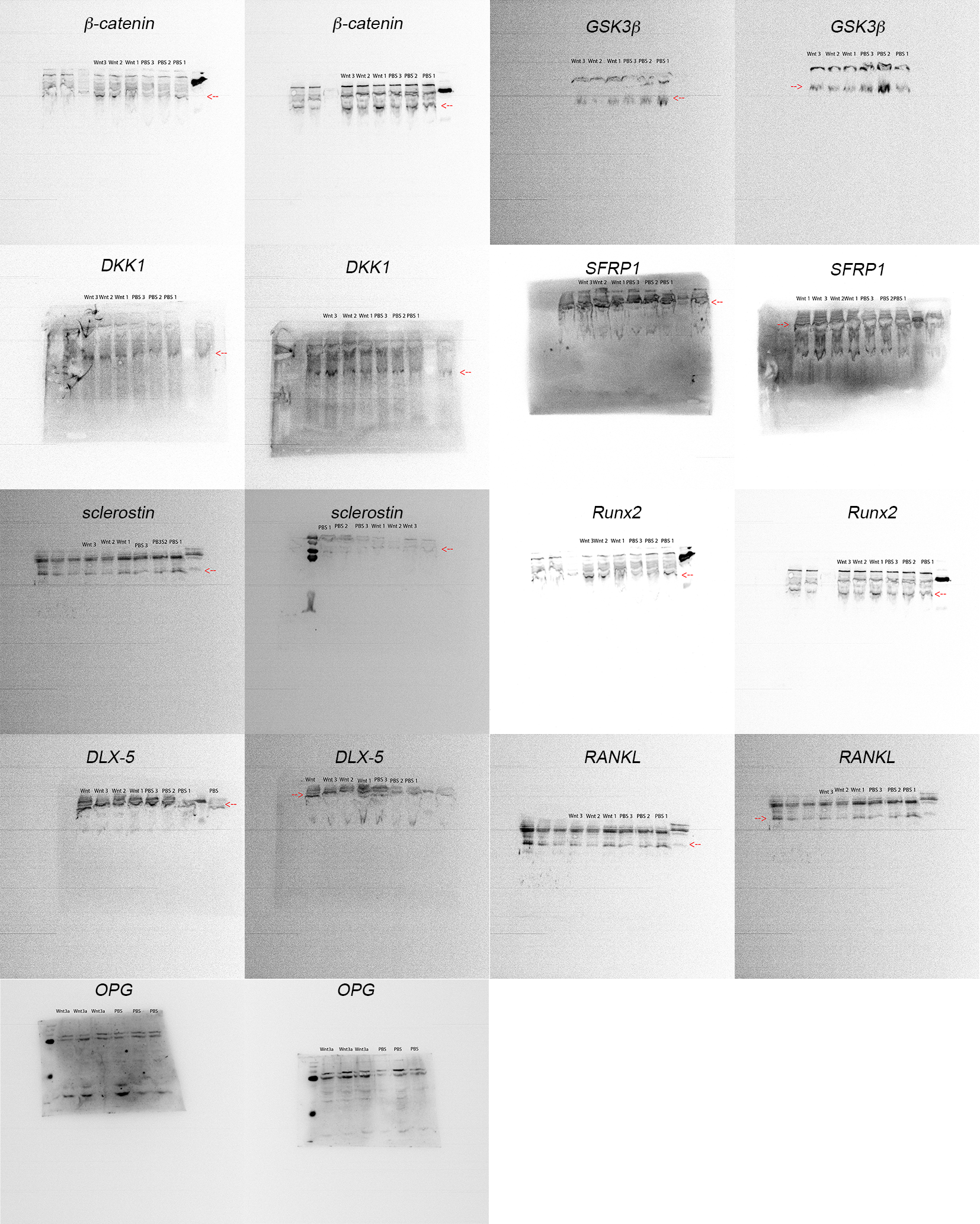

Supplement: Supplementary file 2 — High resolution image (TIF 3484 kb) [file 109_2020_1924_MOESM1_ESM.tif]
